# Entropy Production in a Fractal System with Diffusive Dynamics

**DOI:** 10.3390/e25121578

**Published:** 2023-11-23

**Authors:** Rafael S. Zola, Ervin K. Lenzi, Luciano R. da Silva, Marcelo K. Lenzi

**Affiliations:** 1 Departmento de Física, Universidade Tecnológica Federal do Paraná—Campus de Apucarana, Apucarana 86812-460, PR, Brazil; 2 Departamento de Física, Universidade Estadual de Ponta Grossa, Ponta Grossa 84030-900, PR, Brazil; eklenzi@uepg.br; 3 Departamento de Física, Universidade Federal do Rio Grande do Norte, Natal 59078-900, RN, Brazil; luciano@fisica.ufrn.br; 4 Departamento de Engenharia Química, Universidade Federal do Paraná, Curitiba 81531-980, PR, Brazil; lenzi@ufpr.br

**Keywords:** nonlinear diffusion, generalized entropies, H-theorem, entropy production

## Abstract

We study the entropy production in a fractal system composed of two subsystems, each of which is subjected to an external force. This is achieved by using the H-theorem on the nonlinear Fokker–Planck equations (NFEs) characterizing the diffusing dynamics of each subsystem. In particular, we write a general NFE in terms of Hausdorff derivatives to take into account the metric of each system. We have also investigated some solutions from the analytical and numerical point of view. We demonstrate that each subsystem affects the total entropy and how the diffusive process is anomalous when the fractal nature of the system is considered.

## 1. Introduction

Perhaps one of the greatest achievements of modern physics is the statistical mechanics formalism, first developed by Boltzmann [1] and later expanded by Gibbs [2], to describe the dynamics of microscopic particles and their connection with observable thermodynamics. Entropy is the crucial ingredient in this theory, which must verify the H-Theorem, establishing that nonequilibrium systems will reach equilibrium after long-time evolution, essentially posing a way of investigating the rule of additivity for systems with different entropies [3,4,5,6]. From a microscopic perspective, the Langevin equation is typically employed when studying brownian motion, which results in the time-dependent position of the particle when friction and noise are considered. From the phenomenological point of view, nonequilibrium statistical mechanics, including entropy calculation, is typically calculated with the Fokker–Planck equation which, in its linear form, results in Gaussian distributions, whereas the NFE is generally used to describe anomalous behavior often seen in long-range interaction [7], memory effects [8], porous media [9,10], and many others (see, for example, Refs. [11,12,13] and references therein), all of which display non-Gaussian distributions, typical of non-Markovian characteristics. A remarkable case in which non-Gaussian behavior may be present is in the case of interacting particles/systems. For example, a set of particles may react with another set, which may result in combination, or conversion of one chemical species into another. In this case, as the process occurs, the interaction dynamically affects the physical parameters of the system as a whole, including the entropy production and the diffusion coefficient, which depend on temperature and how the particles interact with each other [14]. Thus, such cases may be seen as a dynamically coupled system [14].

An anomalous process, such as anomalous diffusion, is a burgeoning field of research, as it is often observed across several research fields, from separation to biological media diffusion. From a theoretical point of view, fractional calculus is commonly used to describe anomalous diffusion processes. However, fractional calculus is nonlocal, making it troublesome in the numerical simulation of long-term and large-scale problems [15]. Also, the Mittag–Leffler decay [16] and the Lévy stable statistics [17] resulting from fractional calculus do not describe well some stretched relaxation and stretched Gaussian statistics [15]. An alternative approach, a local differential operator (conformable derivative [18]), is the Hausdorff derivative [19], which relates the fractal nature of space (or time) to the appearance of anomalous behavior such as observed in anomalous diffusion [15]. Fractal derivatives have been applied in several contexts, from anomalous diffusion to viscoelasticity and water transport [15]. The use of Hausdorff derivatives in generalized Fokker–Planck equations is also scarce. For example, recently [20], the solutions for the generalized Fokker–Planck equation with conformable and integro-differential operators were analyzed, indicating that the mean square displacement (MSD) for the studied case presented some difference when compared to the Caputo derivative [21], but presents the same time dependence as the scaled Brownian motion [20]. The Fokker–Planck equation of fractal curves was also explored in reference [22], where anomalous diffusion is observed. Hausdorff derivatives have also been used to describe anomalous transport in porous media by assuming a non-Euclidian fractal metric, displaying better agreement with experimental data for the heavy tail distribution [23].

This article studies the entropy production in a fractal system composed of two subsystems. Hence, this article is devoted to study a system composed of two different set of diffusing particles, each set a subsystem of the whole system. As each subsystem relaxes, the change in one subsystem affects the other, since the time dependent diffusion coefficient of one subsystem has to change as the distribution of the other subsystem evolve, as previously discussed for interacting particles. In fact, it is well known that morphology directly affects diffusion [24,25], so the change in concentration of interacting particles, commonly reported as molecular crowding [26], may be viewed as a morphology change. Aiming to make our model as general as possible, we use a set of NFEs to describe the evolution of each subsystem and analyze different dependences of the time-dependent diffusion coefficient of one subsystem on the distribution of the other. Also, we solve our equations with the Hausdorff metric so each subsystem may be viewed as fractal in nature. Furthermore, we use this system to calculate the entropy production, which is also generalized by assuming different forms for the entropy of each subsystem. The thermodynamic connection is achieved by using the H-Theorem on the NFE equations, characterizing the diffusing dynamics of each subsystem, each subjected to an external force. In this instance, the diffusion coefficient is temperature-dependent, and the entropy is also temperature-dependent, so the behavior of one system affects the other. Consequently, each subsystem serves as a thermal bath for the other, and the H-Theorem guarantees a connection between the subsystems. This feature also helps us to analyze entropy and the zero-law of the thermodynamics in terms of a relaxation process governed by nonlinear Fokker–Planck equations or the mixing between different regimes of diffusion. In fact, the nonlinear or mixing between different terms connected to the relaxation process directly influences the functional entropy form and, consequently, on the properties such as the additivity when different subsystems are added to compose the system. In this sense, the approach considered here gives a suitable form for the entropy and nonlinear Fokker–Planck equations in connection with these processes in a thermostatistic context for usual or anomalous relaxation processes. In this manner, the results will show that the dynamic of each one has a direct influence on the other as a thermal bath since the coupling appears in the diffusive term, which is related to how the system will spread. We express a general NFE regarding Hausdorff derivatives, considering each system’s fractal metric. We show that the fractal order significantly affects entropy production and leads to an anomalous diffusion behavior for each design.

## 2. Nonlinear Fokker–Planck Equations and Hausdorff Derivative

We begin by setting up the NFEs that explain the behavior of each part of a composite system:

(1) $$\begin{array}{ccc} \frac{\partial }{\partial t}\rho_{1}\left(x_{1},t\right) & = & -\frac{\partial }{\partial \xi_{I,1}\left(x_{1}\right)}J_{1}\left(x_{1},t\right) \end{array}$$

(2) $$\begin{array}{ccc} J_{1}\left(x_{1},t\right) & = & -D\frac{\partial }{\partial \xi_{I,1}\left(x_{1}\right)}P_{1}\left(\rho_{1},t\right)+\frac{\partial }{\partial \xi_{I,1}\left(x_{1}\right)}\left[F_{1}\left(x_{1}\right)\rho_{1}\left(x_{1},t\right)\right] \end{array}$$

which implies

(3) $$\begin{array}{c} \frac{\partial }{\partial t}\rho_{1}\left(x_{1},t\right)=\frac{\partial }{\partial \xi_{I,1}\left(x_{1}\right)}\left\{D\frac{\partial }{\partial \xi_{I,1}\left(x_{1}\right)}P_{1}\left(\rho_{1},t\right)-\frac{\partial }{\partial \xi_{I,1}\left(x_{1}\right)}\left[F_{1}\left(x_{1}\right)\rho_{1}\left(x_{1},t\right)\right]\right\} \end{array}$$

and

(4) $$\begin{array}{ccc} \frac{\partial }{\partial t}\rho_{2}\left(x_{2},t\right) & = & -\frac{\partial }{\partial \xi_{I,2}\left(x_{2}\right)}J_{2}\left(x_{2},t\right) \end{array}$$

(5) $$\begin{array}{ccc} J_{2}\left(x_{2},t\right) & = & -D\frac{\partial }{\partial \xi_{I,2}\left(x_{2}\right)}P_{2}\left(\rho_{2},t\right)+\frac{\partial }{\partial \xi_{I,2}\left(x_{2}\right)}\left[F_{2}\left(x_{2}\right)\rho_{2}\left(x_{2},t\right)\right] \end{array}$$

which implies

(6) $$\begin{array}{c} \frac{\partial }{\partial t}\rho_{2}\left(x_{2},t\right)=\frac{\partial }{\partial \xi_{I,2}\left(x_{2}\right)}\left\{D\frac{\partial }{\partial \xi_{I,2}\left(x_{2}\right)}P_{2}\left(\rho_{2},t\right)-\frac{\partial }{\partial \xi_{I,2}\left(x_{2}\right)}\left[F_{2}\left(x_{2}\right)\rho_{2}\left(x_{2},t\right)\right]\right\}. \end{array}$$

In Equations (1)–(6), $\rho_{1(2)}$ is the particle distribution in system 1(2). An external force $F_{i}\left(x_{i}\right)$ is applied to each subsystem, with *i* being 1 or 2. This force is connected to the potential energy $\phi_{i}$ as $F_{i}=-\partial_{\xi_{I,i}\left(x_{i}\right)}\phi_{i}\left(x_{i}\right)$, $x_{1}$ and $x_{2}$ are defined in the range (−∞,∞) where the diffusion proceeds, D is the diffusion coefficient, and the spatial operator is the Hausdorff derivative [15,27], as shall be defined below. Furthermore, $P_{1(2)}\left(\rho_{1(2)},t\right)$ is a functional depending on the distribution of particles here used to generalize the problem. In fact, this research will make use of $P_{1}\left(\rho_{1},t\right)$ and $P_{2}\left(\rho_{2},t\right)$ to illustrate a certain phenomenon, as previously seen in porous media [28], anomalous diffusion [29], overdamped systems [30], and the Boltzmann equation with a correlation term [31]. Equations (3) and (6) also extend the equations used in Refs. [32,33,34,35,36,37] to analyze the H-theorem and the entropy production enable us to consider different contexts. One of them is the relaxation to an equilibrium, a system composed of subsystems that are governed by Equations (3) and (6), which may be connected to the zero law of the thermodynamics in generalized thermostatistics contexts [38,39,40]. The spatial differential operator in Equations (1)–(5), the Hausdorff derivative [15,27], is defined as follows:

(7) $$\begin{array}{c} \frac{\partial }{\partial \xi_{I,i}\left(x_{i}\right)}\rho_{i}\left(x_{i},t\right)=\lim_{x_{i}^{\prime }\to x_{i}}\frac{\rho_{i}\left(x_{i},t\right)-\rho_{i}\left(x_{i}^{\prime },t\right)}{\xi_{I,i}\left(x_{i}\right)-\xi_{I,i}\left(x_{i}^{\prime }\right)}=\frac{1}{\xi_{i}\left(x_{i}\right)}\frac{\partial }{\partial x_{i}}\rho_{i}\left(x_{i},t\right), \end{array}$$

where $\xi_{I,i}\left(x_{i}\right)=\int^{x_{i}}d\bar{x}\xi_{i}\left(\bar{x}\right)$ which, as previously discussed, may be connected to the fractal aspects of each system [19].

### 2.1. H-Theorem

We begin by applying the H-Theorem, taking into account $P_{1}\left(\rho_{1},t\right)$ formally equal to $P_{2}\left(\rho_{2},t\right)$. We will then explore the consequences of making a different selection and how it impacts the entropy of the combined system. Subsequently, we will calculate the Helmholtz free energy and its rate of change, as outlined in Refs. [35,41,42]. The free energy is expressed as F=U−TS, with the internal energy, *U*, given as:

(8) $$\begin{array}{c} U=\int_{-\infty }^{\infty }dx_{1}\xi_{1}\left(x_{1}\right)\int_{-\infty }^{\infty }dx_{2}\xi_{2}\left(x_{2}\right)\Xi \left(x_{1},x_{2}\right)\rho_{1}\left(x_{1},t\right)\rho_{2}\left(x_{2},t\right), \end{array}$$

with $\Xi \left(x_{1},x_{2}\right)=\phi_{1}\left(x_{1}\right)+\phi_{2}\left(x_{2}\right)$, and the entropy *S* calculated as:

(9) $$\begin{array}{c} S=k\int_{-\infty }^{\infty }dx_{1}\xi_{1}\left(x_{1}\right)\int_{-\infty }^{\infty }dx_{2}\xi_{2}\left(x_{2}\right)s\left(\rho_{1},\rho_{2}\right) \end{array}$$

where $s(\rho_{1},\rho_{2})$ represents a generalized entropy form. For example, if $s(\rho_{1},\rho_{2})$=$\rho_{1}\ln\rho_{1}$+$\rho_{2}\ln\rho_{2}$, we recover the classical Boltzmann entropy. The total free energy of the system composed of two subsystems is expressed by Equations (8) and (9).

(10) $$\begin{array}{c} F=\int_{-\infty }^{\infty }dx_{1}\xi_{1}\left(x_{1}\right)\int_{-\infty }^{\infty }dx_{2}\xi_{2}\left(x_{2}\right)[\Xi \left(x_{1},x_{2}\right)\rho_{1}\left(x_{1},t\right)\rho_{2}\left(x_{2},t\right)-kTs\left(\rho_{1},\rho_{2}\right)]. \end{array}$$

Before examining the H-Theorem with Equation (10), we will assume that $P_{1}\left(\rho_{1},t\right)$ and $P_{2}\left(\rho_{2},t\right)$ have the same structure and that the entropy is a function of the product of the probability densities of each subsystem, i.e., $s\left(\rho_{1},\rho_{2}\right)=s\left(\rho_{1}\rho_{2}\right)$. This allows us to demonstrate that

(11) $$\begin{array}{c} \frac{d}{dt}F=\int_{-\infty }^{\infty }dx_{1}\xi_{1}\left(x_{1}\right)\int_{-\infty }^{\infty }dx_{2}\xi_{2}\left(x_{2}\right)\left[\Xi \left(x_{1},x_{2}\right)-kT\frac{\partial }{\partial \rho_{12}}s\left(\rho_{12}\right)\right]\frac{\partial }{\partial t}\left[\rho_{1}\left(x_{1},t\right)\rho_{2}\left(x_{2},t\right)\right], \end{array}$$

in which $\rho_{12}=\rho_{1}\rho_{2}$. By performing some calculations and using Equations (2) and (5), we have that

(12) $$\begin{array}{ccc} \frac{d}{dt}F & = & \int_{-\infty }^{\infty }dx_{1}\xi_{1}\left(x_{1}\right)\int_{-\infty }^{\infty }dx_{2}\xi_{2}\left(x_{2}\right)\left\{\Xi \left(x_{1},x_{2}\right)\rho_{2}-kT\rho_{2}\frac{\partial }{\partial \rho_{12}}s\left(\rho_{12}\right)\right\}\\ & \times & \frac{\partial }{\partial \xi_{I,1}\left(x_{1}\right)}\left\{D\frac{\partial }{\partial \xi_{I,1}\left(x_{1}\right)}P_{1}\left(\rho_{1},t\right)-\frac{\partial }{\partial \xi_{I,1}\left(x_{1}\right)}\left[F_{1}\left(x_{1}\right)\rho_{1}\left(x_{1},t\right)\right]\right\}\\ & + & \int_{-\infty }^{\infty }dx_{1}\xi_{1}\left(x_{1}\right)\int_{-\infty }^{\infty }dx_{2}\xi_{2}\left(x_{2}\right)\left\{\Xi \left(x_{1},x_{2}\right)\rho_{1}-kT\rho_{1}\frac{\partial }{\partial \rho_{12}}s\left(\rho_{12}\right)\right\}\\ & \times & \frac{\partial }{\partial \xi_{I,2}\left(x_{2}\right)}\left\{D\frac{\partial }{\partial \xi_{I,2}\left(x_{2}\right)}P_{2}\left(\rho_{2},t\right)-\frac{\partial }{\partial \xi_{I,2}\left(x_{2}\right)}\left[F_{2}\left(x_{2}\right)\rho_{2}\left(x_{2},t\right)\right]\right\}. \end{array}$$

We now assume the following conditions: $\rho_{i}\left(x_{i}\to \pm \infty ,t\right)\to 0$ and $\partial_{\xi_{I,i}\left(x_{i}\right)}\rho_{i}\left(x_{i}\to \pm \infty ,t\right)$→ 0. Thus, Equation (12) becomes, after integration by parts:

(13) $$\begin{array}{ccc} \frac{d}{dt}F & = & -\int_{-\infty }^{\infty }dx_{1}\xi_{1}\left(x_{1}\right)\int_{-\infty }^{\infty }dx_{2}\xi_{2}\left(x_{2}\right)\left\{\frac{\partial \phi_{1}\left(x_{1}\right)}{\partial \xi_{I,1}\left(x_{1}\right)}\rho_{2}-kT\rho_{2}^{2}\frac{\partial \rho_{1}}{\partial \xi_{I,1}\left(x_{1}\right)}\frac{\partial^{2}}{\partial \rho_{12}^{2}}s\left(\rho_{12}\right)\right\}\\ & \times & \left\{D\frac{\partial }{\partial \xi_{I,1}\left(x_{1}\right)}P_{1}\left(\rho_{1},t\right)+\frac{\partial \phi_{1}\left(x_{1}\right)}{\partial \xi_{I,1}\left(x_{1}\right)}\rho_{1}\left(x_{1},t\right)\right\}\\ & - & \int_{-\infty }^{\infty }dx_{1}\xi_{1}\left(x_{1}\right)\int_{-\infty }^{\infty }dx_{2}\xi_{2}\left(x_{2}\right)\left\{\frac{\partial \phi_{2}\left(x_{2}\right)}{\partial \xi_{I,2}\left(x_{2}\right)}\rho_{1}-kT\rho_{1}^{2}\frac{\partial \rho_{2}}{\partial \xi_{I,2}\left(x_{2}\right)}\frac{\partial^{2}}{\partial \rho_{12}^{2}}s\left(\rho_{12}\right)\right\}\\ & \times & \left\{D\frac{\partial }{\partial \xi_{I,2}\left(x_{2}\right)}P_{2}\left(\rho_{2},t\right)+\frac{\partial \phi_{2}\left(x_{2}\right)}{\partial \xi_{I,2}\left(x_{2}\right)}\rho_{2}\left(x_{2},t\right)\right\} \end{array}$$

(for more details, see the Appendix A). Equation (13) is a general result and can be connected to different relaxation processes depending on the choice of $P_{1}\left(\rho_{1},t\right)$ and $P_{2}\left(\rho_{2},t\right)$ present in Equations (3) and (6). It can also be used to evidence the interaction between the subsystems in connection with the relaxation process each subsystem exhibits. This feature will be evident below with the analysis of the condition required for the H-Theorem and the previous assumption for the entropy, i.e., $s\left(\rho_{1},\rho_{2}\right)=s\left(\rho_{1}\rho_{2}\right)$. Also, this result maintains the additivity in Penrose sense [43], i.e., $S\left(\rho_{12}\right)=S\left(\rho_{1}\rho_{2}\right)$ required for a system composed of independent subsystems when the standard entropy is employed. Thus, the conditions required by Equation (13) to verify the H-theorem will define a suitable entropy for the relaxation process described in terms of Equations (3) and (6) for a choice of $P_{1}\left(\rho_{1},t\right)$ and $P_{2}\left(\rho_{2},t\right)$, and consequently, the properties of this entropy in connection with a thermostatistical context.

To proceed with our analysis, we consider that

(14) $$\begin{array}{c} P_{i}\left(\rho_{i},t\right)=\int_{0}^{\gamma }d\bar{\gamma }p\left(\bar{\gamma }\right)D_{j,\gamma }\left(t\right)\rho_{i}^{\bar{\gamma }}\left(x_{i},t\right), \end{array}$$

with j≠i, $p(\bar{\gamma })$ is a distribution, and $D_{j,\bar{\gamma }}\left(t\right)=\int_{-\infty }^{\infty }dx_{j}\xi_{j}\left(x_{j}\right)\rho_{j}^{\bar{\gamma }}\left(x_{j},t\right)$. Note that the distribution $p(\bar{\gamma })$ is connected with the nonlinear term present in Equations (3) and (6), which leads us a diffusive term with different diffusion regimes. In connection with the porous media equation and the Tsallis framework [11], $\bar{\gamma }$ may be related to the nonlinearity present in Equations (3) and (6) after substituting Equation (14) and also with the extension of the entropy to accommodate a thermostatistic context. The range of γ is connected with the choice performed for the distribution $p(\bar{\gamma })$, which defines how will be the behavior of the diffusive term. One possibility will be analyzed later by considering two different regimes of diffusion, with $p\left(\bar{\gamma }\right)=\delta \left(\bar{\gamma }-1\right)/2+\delta \left(\bar{\gamma }-\nu \right)/2$, with max(1,ν)≤γ. The choice performed for $D_{j,\bar{\gamma }}\left(t\right)$ also implies that each subsystem influences the other, i.e., Equations (2) and (5) are coupled by the diffusive term. Thus, $D_{j,\bar{\gamma }}\left(t\right)$ introduces interactions between the subsystems during the thermalization process, where each subsystem works as an additional thermal bath to the other. By substituting Equation (14) into Equation (13), we have that

(15) $$\begin{array}{ccc} \;\;\;\;\;\;\frac{d}{dt}F\;\;\; & = & \;\;\;-\int_{-\infty }^{\infty }dx_{1}\xi_{1}\left(x_{1}\right)\frac{1}{\rho_{1}\left(x_{1}\right)}\\ & \times & \;\;\;\left\{\int_{-\infty }^{\infty }dx_{2}\xi_{2}\left(x_{2}\right)\left[\frac{\partial \phi_{1}\left(x_{1}\right)}{\partial \xi_{I,1}\left(x_{1}\right)}\rho_{2}\rho_{1}-kT\rho_{2}^{2}\rho_{1}\frac{\partial \rho_{1}\left(x_{1}\right)}{\partial \xi_{I,1}\left(x_{1}\right)}\frac{\partial^{2}}{\partial \rho_{12}^{2}}s\left(\rho_{12}\right)\right]\right.\\ & \times & \;\;\;\left.\int_{-\infty }^{\infty }dx_{2}\xi_{2}\left(x_{2}\right)\left[\frac{\partial \phi_{1}\left(x_{1}\right)}{\partial \xi_{I,1}\left(x_{1}\right)}\rho_{1}\rho_{2}+D\frac{\partial \rho_{1}\left(x_{1}\right)}{\partial \xi_{I,1}\left(x_{1}\right)}\rho_{2}\frac{\partial }{\partial \rho_{12}}\left(\int_{0}^{\gamma }d\bar{\gamma }p\left(\bar{\gamma }\right)\rho_{2}^{\bar{\gamma }}\rho_{1}^{\bar{\gamma }}\right)\right]\right\}\\ & - & \;\;\;\int_{-\infty }^{\infty }dx_{2}\xi_{2}\left(x_{2}\right)\frac{1}{\rho_{2}\left(x_{2}\right)}\\ & \times & \left\{\int_{-\infty }^{\infty }dx_{1}\xi_{1}\left(x_{1}\right)\left[\frac{\partial \phi_{2}\left(x_{2}\right)}{\partial \xi_{I,2}\left(x_{2}\right)}\rho_{1}\rho_{2}-kT\rho_{1}^{2}\rho_{2}\frac{\partial \rho_{2}\left(x_{2}\right)}{\partial \xi_{I,2}\left(x_{2}\right)}\frac{\partial^{2}}{\partial \rho_{12}^{2}}s\left(\rho_{12}\right)\right]\right.\\ & \times & \;\;\;\int_{-\infty }^{\infty }dx_{1}\xi_{1}\left(x_{1}\right)\left.\left[\frac{\partial \phi_{2}\left(x_{2}\right)}{\partial \xi_{I,2}\left(x_{2}\right)}\rho_{1}\rho_{2}+D\frac{\partial \rho_{2}\left(x_{2}\right)}{\partial \xi_{I,2}\left(x_{2}\right)}\rho_{1}\frac{\partial }{\partial \rho_{12}}\left(\int_{0}^{\gamma }d\bar{\gamma }p\left(\bar{\gamma }\right)\rho_{2}^{\bar{\gamma }}\rho_{1}^{\bar{\gamma }}\right)\right]\right\}. \end{array}$$

We verify that

(16) $$\begin{array}{ccc} \frac{d}{dt}F\leq 0\;\;\mathrm{if}\;\;-kT\rho_{j}^{2}\rho_{i}\frac{\partial^{2}}{\partial \rho_{ij}^{2}}s\left(\rho_{ij}\right) & = & D\rho_{j}\frac{\partial }{\partial \rho_{ij}}\left(\int_{0}^{\gamma }d\bar{\gamma }p\left(\bar{\gamma }\right)\rho_{i}^{\bar{\gamma }}\rho_{j}^{\bar{\gamma }}\right)\;, \end{array}$$

(17) $$\begin{array}{ccc} & = & D\rho_{j}\frac{\partial }{\partial \rho_{ij}}\left(\int_{0}^{\gamma }d\bar{\gamma }p\left(\bar{\gamma }\right)\rho_{ij}^{\bar{\gamma }}\right)\;, \end{array}$$

for i=1,2 and j=1,2 with i≠j, D=kT, and $\rho_{ij}=\rho_{i}\rho_{j}$, which implies

(18) $$\begin{array}{ccc} \frac{d}{dt}F & = & -\int_{-\infty }^{\infty }dx_{1}\xi_{1}\left(x_{1}\right)\frac{1}{\rho_{1}\left(x_{1}\right)}\\ & \times & {\left\{\int_{-\infty }^{\infty }dx_{2}\xi_{2}\left(x_{2}\right)\left[\frac{\partial \phi_{1}\left(x_{1}\right)}{\partial \xi_{I,1}\left(x_{1}\right)}\rho_{2}\rho_{1}-kT\rho_{2}^{2}\rho_{1}\frac{\partial \rho_{1}}{\partial \xi_{I,1}\left(x_{1}\right)}\frac{\partial^{2}}{\partial \rho_{12}^{2}}s\left(\rho_{12}\right)\right]\right\}}^{2}\\ & - & \int_{-\infty }^{\infty }dx_{2}\xi_{2}\left(x_{2}\right)\frac{1}{\rho_{2}\left(x_{2}\right)}\\ & \times & {\left\{\int_{-\infty }^{\infty }dx_{1}\xi_{1}\left(x_{1}\right)\left[\frac{\partial \phi_{2}\left(x_{2}\right)}{\partial \xi_{I,2}\left(x_{2}\right)}\rho_{1}\rho_{2}-kT\rho_{1}^{2}\rho_{2}\frac{\partial \rho_{2}}{\partial \xi_{I,2}\left(x_{2}\right)}\frac{\partial^{2}}{\partial \rho_{12}^{2}}s\left(\rho_{12}\right)\right]\right\}}^{2}. \end{array}$$

By solving Equation (16) under the conditions defined in Refs. [34,35,41,42], we obtain

(19) $$\begin{array}{c} s\left(\rho_{12}\right)=\int_{0}^{\gamma }d\bar{\gamma }p\left(\bar{\gamma }\right)\frac{1}{\bar{\gamma }-1}\left(\rho_{12}-\rho_{12}^{\bar{\gamma }}\right). \end{array}$$

The entropy for the composite system is given by

(20) $$\begin{array}{c} S=k\int_{-\infty }^{\infty }dx_{1}\xi_{1}\left(x_{1}\right)\int_{-\infty }^{\infty }dx_{2}\xi_{2}\left(x_{2}\right)\int_{0}^{\gamma }d\bar{\gamma }p\left(\bar{\gamma }\right)\frac{1}{\bar{\gamma }-1}\left(\rho_{12}-\rho_{12}^{\bar{\gamma }}\right), \end{array}$$

which can also be rewritten as

(21) $$\begin{array}{c} S=k\int_{-\infty }^{\infty }dx_{1}\xi_{1}\left(x_{1}\right)\int_{-\infty }^{\infty }dx_{2}\xi_{2}\left(x_{2}\right)\int_{0}^{\gamma }d\bar{\gamma }p\left(\bar{\gamma }\right)\frac{1}{\bar{\gamma }-1}\left(\rho_{1}\rho_{2}-{\left(\rho_{1}\rho_{2}\right)}^{\bar{\gamma }}\right) \end{array}$$

and, consequently, as

(22) $$\begin{array}{c} S=k\int_{0}^{\gamma }d\bar{\gamma }p\left(\bar{\gamma }\right)\frac{1}{\bar{\gamma }-1}\left(1-\int_{-\infty }^{\infty }dx_{1}\xi_{1}\left(x_{1}\right)\int_{-\infty }^{\infty }dx_{2}\xi_{2}\left(x_{2}\right){\left[\rho_{1}\left(x_{1}\right)\rho_{2}\left(x_{2}\right)\right]}^{\bar{\gamma }}\right). \end{array}$$

Equation (22) may have several particular cases, such as the Tsallis and Kaniadakis entropies, depending on the choice of $p(\bar{\gamma })$. It is also worth mentioning that the Boltzmann–Gibbs entropy is recovered from Equation (22), which is connected with the linear Fokker–Planck equation as discussed in Ref. [44]. In addition, different from the standard situation, to satisfy the H-theorem and preserve the form $s=s(\rho_{1}\rho_{2})$, the nonlinear Fokker–Planck equations imply that the systems interact with each other. Equation (22) can be connected to the unusual additivity present in the Tsallis formalism as follows:

(23) $$\begin{array}{c} S=\int_{0}^{\gamma }d\bar{\gamma }p\left(\bar{\gamma }\right)(S_{1}\left(\bar{\gamma }\right)+S_{2}\left(\bar{\gamma }\right)+\left[\left(1-\bar{\gamma }\right)/k\right]S_{1}\left(\bar{\gamma }\right)S_{2}\left(\bar{\gamma }\right))\;, \end{array}$$

where

(24) $$\begin{array}{c} S_{1(2)}\left(\bar{\gamma }\right)=\frac{k}{\bar{\gamma }-1}\left(1-\int_{-\infty }^{\infty }dx_{1(2)}\xi_{1(2)}\left(x_{1(2)}\right){\left[\rho_{1(2)}\left(x_{1(2)}\right)\right]}^{\bar{\gamma }}\right). \end{array}$$

This result, connected to each system’s relaxation, shows how each part’s entropy is added to compose the total entropy. Equation (23) has been applied in several situations such as black hole [45], inanimated and living matter [46], and interacting particles [47].

The NFE that emerges from the previous analysis, i.e., from Equation (14), which is related to the nonlinear term and verifies the H-theorem, for each subsystem can be written as follows:

(25) $$\begin{array}{c} \frac{\partial }{\partial t}\rho_{1}\left(x_{1},t\right)=\frac{\partial }{\partial \xi_{I,1}\left(x_{1}\right)}\left\{\int_{0}^{\gamma }d\bar{\gamma }p\left(\bar{\gamma }\right){\bar{D}}_{1,\bar{\gamma }}\left(t\right)\frac{\partial }{\partial \xi_{I,1}\left(x_{1}\right)}\rho_{1}^{\bar{\gamma }}\left(x_{1},t\right)-F_{1}\left(x_{1}\right)\rho_{1}\left(x_{1},t\right)\right\} \end{array}$$

and

(26) $$\begin{array}{c} \frac{\partial }{\partial t}\rho_{2}\left(x_{2},t\right)=\frac{\partial }{\partial \xi_{I,2}\left(x_{2}\right)}\left\{\int_{0}^{\gamma }d\bar{\gamma }p\left(\bar{\gamma }\right){\bar{D}}_{2,\bar{\gamma }}\left(t\right)\frac{\partial }{\partial \xi_{I,2}\left(x_{2}\right)}\rho_{2}^{\bar{\gamma }}\left(x_{2},t\right)-F_{2}\left(x_{2}\right)\rho_{2}\left(x_{2},t\right)\right\}, \end{array}$$

with ${\bar{D}}_{i,\bar{\gamma }}\left(t\right)=D_{i,\gamma }\left(t\right)D$, which demonstrates the influence of one of the subsystems on the other. In addition, the components that constitute the diffusive element can also be associated with anomalous diffusion processes with distinct diffusion regimes. This feature was investigated, for example, in Ref. [48] by considering the Fokker–Planck equation with a different form for the diffusive term or Langevin equations with additive noises in Ref. [49]. It is also worth mentioning that the H-theorem shows us a suitable way for the dynamic processes in connection with the system’s entropy.

We will now contemplate a general situation in which the diffusion terms have a distinct nonlinear relationship with the distributions. This implies that the systems have distinct dynamic characteristics regulated by the nonlinear connection with the distribution found in the diffusive term. Using the preceding equations and having in mind Equation (10), we may write

(27) $$\begin{array}{ccc} \frac{d}{dt}F & = & \int_{-\infty }^{\infty }dx_{1}\xi_{1}\left(x_{1}\right)\int_{-\infty }^{\infty }dx_{2}\xi_{2}\left(x_{2}\right)\left\{\Xi \left(x_{1},x_{2}\right)\frac{\partial }{\partial t}\left[\rho_{1}\left(x_{1},t\right)\rho_{2}\left(x_{2},t\right)\right]\right.\\ & - & \left.kT\left[\frac{\partial }{\partial \rho_{1}}s\left(\rho_{1},\rho_{2}\right)\frac{\partial }{\partial t}\rho_{1}\left(x_{1},t\right)+\frac{\partial }{\partial \rho_{2}}s\left(\rho_{1},\rho_{2}\right)\frac{\partial }{\partial t}\rho_{2}\left(x_{2},t\right)\right]\right\}, \end{array}$$

which implies

(28) $$\begin{array}{ccc} \frac{d}{dt}F & = & \int_{-\infty }^{\infty }dx_{1}\xi_{1}\left(x_{1}\right)\int_{-\infty }^{\infty }dx_{2}\xi_{2}\left(x_{2}\right)\left\{\Xi \left(x_{1},x_{2}\right)\rho_{2}-kT\frac{\partial }{\partial \rho_{1}}s\left(\rho_{1},\rho_{2}\right)\right\}\\ & \times & \frac{\partial }{\partial \xi_{I,1}\left(x_{1}\right)}\left\{D\frac{\partial }{\partial \xi_{I,1}\left(x_{1}\right)}P_{1}\left(\rho_{1},t\right)+\frac{\partial \phi_{1}\left(x_{1}\right)}{\partial \xi_{I,1}\left(x_{1}\right)}\rho_{1}\left(x_{1},t\right)\right\}\\ & + & \int_{-\infty }^{\infty }dx_{1}\xi_{1}\left(x_{1}\right)\int_{-\infty }^{\infty }dx_{2}\xi_{2}\left(x_{2}\right)\left\{\Xi \left(x_{1},x_{2}\right)\rho_{1}-kT\frac{\partial }{\partial \rho_{2}}s\left(\rho_{1},\rho_{2}\right)\right\}\\ & \times & \frac{\partial }{\partial \xi_{I,2}\left(x_{2}\right)}\left\{D\frac{\partial }{\partial \xi_{I,2}\left(x_{2}\right)}P_{2}\left(\rho_{2},t\right)+\frac{\partial \phi_{2}\left(x_{2}\right)}{\partial \xi_{I,2}\left(x_{2}\right)}\rho_{2}\left(x_{2},t\right)\right\}. \end{array}$$

After some calculations, it is possible to show that

(29) $$\begin{array}{ccc} \frac{d}{dt}F & = & -\int_{-\infty }^{\infty }dx_{1}\xi_{1}\left(x_{1}\right)\frac{1}{\rho_{1}\left(x_{1}\right)}\int_{-\infty }^{\infty }dx_{2}\xi_{2}\left(x_{2}\right)\\ & \times & \left\{\frac{\partial \phi (x_{1})}{\partial \xi_{I,1}\left(x_{1}\right)}\rho_{2}\left(x_{2}\right)\rho_{1}\left(x_{1}\right)-kT\left[\rho_{1}\frac{\partial^{2}}{\partial \rho_{1}^{2}}s\left(\rho_{1},\rho_{2}\right)\right]\frac{\partial \rho_{1}}{\partial \xi_{I,1}\left(x_{1}\right)}\right\}\\ & \times & \left\{\left[D\frac{\partial }{\partial \rho_{1}}P_{1}\left(\rho_{1},t\right)\right]\frac{\partial \rho_{1}}{\partial \xi_{I,1}\left(x_{1}\right)}+\frac{\partial \phi_{1}\left(x_{1}\right)}{\partial \xi_{I,1}\left(x_{1}\right)}\rho_{1}\left(x_{1},t\right)\right\}\\ & - & \int_{-\infty }^{\infty }dx_{1}\xi_{1}\left(x_{1}\right)\int_{-\infty }^{\infty }dx_{2}\xi_{2}\left(x_{2}\right)\\ & \times & \frac{1}{\rho_{2}\left(x_{2}\right)}\left\{\frac{\partial \phi (x_{2})}{\partial \xi_{I,2}\left(x_{2}\right)}\rho_{1}\left(x_{1}\right)\rho_{2}\left(x_{2}\right)-kT\left[\rho_{2}\frac{\partial^{2}}{\partial \rho_{2}^{2}}s\left(\rho_{1},\rho_{2}\right)\right]\frac{\partial \rho_{2}}{\partial \xi_{I,2}\left(x_{2}\right)}\right\}\\ & \times & \frac{\partial }{\partial \xi_{I,2}\left(x_{2}\right)}\left\{\left[D\frac{\partial }{\partial \rho_{2}}P_{2}\left(\rho_{2},t\right)\right]\frac{\partial \rho_{2}}{\partial \xi_{I,2}\left(x_{2}\right)}+\frac{\partial \phi_{2}\left(x_{2}\right)}{\partial \xi_{I,2}\left(x_{2}\right)}\rho_{2}\left(x_{2},t\right)\right\}. \end{array}$$

Now, we assume, for example, the case

(30) $$\begin{array}{c} P_{1}\left(\rho_{1},t\right)=\int_{0}^{\gamma }d\bar{\gamma }p\left(\bar{\gamma }\right)D_{2,\nu }\left(t\right)\rho_{1}^{\bar{\gamma }}\left(x_{1},t\right) \end{array}$$

and

(31) $$\begin{array}{c} P_{2}\left(\rho_{2},t\right)=\int_{0}^{\nu }d\bar{\nu }p\left(\bar{\nu }\right)D_{1,\bar{\gamma }}\left(t\right)\rho_{2}^{\bar{\nu }}\left(x_{2},t\right), \end{array}$$

with

(32) $$\begin{array}{c} D_{2,\nu }\left(t\right)=\int_{0}^{\nu }d\bar{\nu }p\left(\bar{\nu }\right)\frac{1}{\bar{\nu }-1}\int_{-\infty }^{\infty }dx_{2}\xi_{2}\left(x_{2}\right)\rho_{2}^{\bar{\nu }}\left(x_{2},t\right) \end{array}$$

and

(33) $$\begin{array}{c} \;\;\;D_{1,\gamma }\left(t\right)=\int_{0}^{\gamma }d\bar{\gamma }p\left(\bar{\gamma }\right)\frac{1}{\bar{\gamma }-1}\int_{-\infty }^{\infty }dx_{1}\xi_{1}\left(x_{1}\right)\rho_{1}^{\bar{\gamma }}\left(x_{1},t\right). \end{array}$$

This implies that Equations (2) and (5) have different forms, and thus, the two subsystems have distinct relaxation processes. In Ref. [14], a particular case was studied by looking at the interaction between the two subsystems. Each choice has its implications for the total entropy of the composite system. By examining Equations (32) and (33), we can see that the entropy must satisfy the following equations:

(34) $$\begin{array}{c} -\rho_{1}\frac{\partial^{2}}{\partial \rho_{1}^{2}}s\left(\rho_{1},\rho_{2}\right)=\int_{0}^{\gamma }d\bar{\gamma }p\left(\bar{\gamma }\right)\int_{0}^{\nu }d\bar{\nu }p\left(\bar{\nu }\right)\frac{\bar{\gamma }}{\bar{\nu }-1}\rho_{2}^{\bar{\nu }}\rho_{1}^{\bar{\gamma }-1} \end{array}$$

and

(35) $$\begin{array}{c} -\rho_{2}\frac{\partial^{2}}{\partial \rho_{2}^{2}}s\left(\rho_{1},\rho_{2}\right)=\int_{0}^{\gamma }d\bar{\gamma }p\left(\bar{\gamma }\right)\int_{0}^{\nu }d\bar{\nu }p\left(\bar{\nu }\right)\frac{\bar{\nu }}{\bar{\gamma }-1}\rho_{2}^{\bar{\nu }-1}\rho_{1}^{\bar{\gamma }} \end{array}$$

to verify

(36) $$\begin{array}{c} \frac{d}{dt}F\leq 0, \end{array}$$

and, consequently, to satisfy the H-Theorem. A solution for the previous system of equations is

(37) $$\begin{array}{c} s\left(\rho_{1},\rho_{2}\right)=\int_{0}^{\gamma }d\bar{\gamma }\frac{p(\bar{\gamma })}{\bar{\gamma }-1}\int_{0}^{\nu }d\bar{\nu }\frac{p(\bar{\nu })}{\bar{\gamma }-1}\left(\rho_{1}\rho_{2}-\rho_{2}^{\bar{\nu }}\rho_{1}^{\bar{\gamma }}\right). \end{array}$$

This result allows us to write the total entropy of this system as follows:

(38) $$\begin{array}{c} S=k\int_{0}^{\gamma }d\bar{\gamma }\frac{p(\bar{\gamma })}{\bar{\gamma }-1}\int_{0}^{\nu }d\bar{\nu }\frac{p(\bar{\nu })}{\bar{\nu }-1}\left[1-\int_{-\infty }^{\infty }dx_{2}\xi_{2}\left(x_{2}\right)\rho_{2}^{\bar{\nu }}\left(x_{2},t\right)\int_{-\infty }^{\infty }dx_{1}\xi_{1}\left(x_{1}\right)\rho_{1}^{\bar{\gamma }}\left(x_{1},t\right)\right]. \end{array}$$

This result for the entropy is distinct from the one given by Equation (22), which was derived from a different selection of NFEs. It is the consequence of combining subsystems with distinct relaxation processes, each of which has its entropy. Additionally, Equation (38) is linked to the combination of Tsallis entropies with different q− indices [50,51,52]. The solution can be obtained in this context by using q− exponential functions.

Let us now consider a particular example of the results mentioned above when different behaviors for $P_{1}\left(\rho_{1},t\right)$ and $P_{2}\left(\rho_{2},t\right)$ are chosen. We consider the case obtained from Equations (30) and (31) for $p\left(\bar{\gamma }\right)=\delta \left(\bar{\gamma }-\gamma \right)$ and $p\left(\bar{\nu }\right)=\delta \left(\bar{\nu }-\nu \right)$ in absence of external force, yielding

(39) $$\begin{array}{c} \frac{\partial }{\partial t}\rho_{1}=\frac{\partial }{\partial \xi_{I,1}\left(x_{1}\right)}\left[D_{2,\nu }\left(t\right)\frac{\partial }{\partial \xi_{I,1}\left(x_{1}\right)}\rho_{1}^{\gamma }\left(x_{1},t\right)\right] \end{array}$$

and

(40) $$\begin{array}{c} \frac{\partial }{\partial t}\rho_{2}=\frac{\partial }{\partial \xi_{I,2}\left(x_{2}\right)}\left[D_{1,\gamma }\left(t\right)\frac{\partial }{\partial \xi_{I,2}\left(x_{2}\right)}\rho_{2}^{\nu }\left(x_{2},t\right)\right]\;. \end{array}$$

Hence, it is simple to verify that

(41) $$\begin{array}{c} \rho_{1}\left(x_{1},t\right)=\exp_{\gamma }\left[-\beta_{1}\left(t\right)\xi_{I,1}^{2}\left(x_{1}\right)\right]/Z_{1}\left(t\right) \end{array}$$

and

(42) $$\begin{array}{c} \rho_{2}\left(x_{1},t\right)=\exp_{\nu }\left[-\beta_{2}\left(t\right)\xi_{I,2}^{2}\left(x_{2}\right)\right]/Z_{2}\left(t\right), \end{array}$$

are solutions for Equations (39) and (40). The previous solutions for the NFEs can be also obtained from the maximum principle of entropy when the entropy

(43) $$\begin{array}{c} S_{1(2)}=\frac{k}{\gamma (\nu )-1}\left(1-\int dx_{1(2)}\xi_{1(2)}\left(x_{1(2)}\right){\left[\rho_{1(2)}\left(x_{1(2)},t\right)\right]}^{\gamma (\nu )}\right) \end{array}$$

with the constraints

(44) $$\begin{array}{c} \int_{-\infty }^{\infty }dx_{1(2)}\xi_{1(2)}\left(x_{1(2)}\right)\rho_{1(2)}^{\gamma (\nu )}\left(x_{1(2)},t\right){\left[\xi_{I,1(2)}\left(x_{1(2)}\right)\right]}^{2}=\sigma_{\xi_{I}\left(x\right),1\left(2\right)}^{2}\left(t\right) \end{array}$$

and

(45) $$\begin{array}{c} \int_{-\infty }^{\infty }dx_{1(2)}\xi_{1(2)}\left(x_{1(2)}\right)\rho_{1(2)}\left(x_{1(2)},t\right)=1\;. \end{array}$$

**Figure 1:**
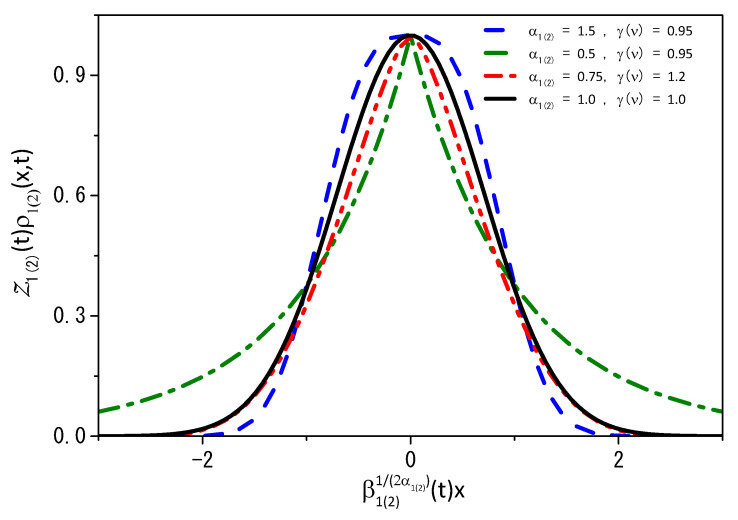
Figure 1 shows the behavior of particle distribution obtained from $Z_{1(2)}\left(t\right)\rho_{1(2)}\left(x,t\right)$ versus $\beta_{1(2)}^{1/\alpha_{1(2)}}\left(t\right)x$ obtained from Equations (41) and (42) for different values of $\alpha_{1(2)}$, ν, and γ. The solid black line corresponds to the standard diffusion, and the other lines correspond to the generalized cases. To make things simpler, we assume $\rho_{1(2)}\left(x_{1(2)},0\right)=\delta \left(x_{1(2)}\right)$, $\xi_{I,1(2)}\left(x_{1(2)}\right)={\left|x_{1(2)}\right|}^{\alpha_{1(2)}}$, and D=1.

A particular situation was worked out in Ref. [11] for the porous media equation. From Equations (41) and (42), we can verify how the distribution is affected by the space metric and the parameter γ(ν). Figure 1 shows $\rho_{1(2)}Z_{1(2)}$ vs. $\beta_{1(2)}x$ for $\xi_{I,1(2)}\left(x_{1(2)}\right)={\left|x_{1(2)}\right|}^{\alpha_{1(2)}}$. If $\alpha_{1(2)}=\gamma \left(\nu \right)=1$, as expected, we recover the usual behavior given by a Gaussian distribution. On the other hand, one can clearly see from figure Figure 1 that if the parameter coming from the Hausdorff derivative ($\alpha_{1(2)}$) or the parameter γ (which describes how the distribution of one kind of particle affects the time-dependent diffusion coefficient of the other, or, in different words, how molecular crowding affects diffusion) the behavior becomes non-Gaussian. Thus, depending on the choice of parameters ν, γ, and $\alpha_{1(2)}$, the distribution may present different behaviors that are asymptotically characterized by short- or long-tailed distributions. In the latter case, it is possible to connect the results with the Lévy distributions. In addition, a particular scenario of Equations (39) and (40) has been worked out in Ref. [53] in connection with the fractal dimensions. Similar situations are found in the context of the Tsallis statistics, which are described by power-law distributions. However, small changes in the parameters $\alpha_{1(2)}$ and γ(ν) strongly affect the distribution of particles.

With $\beta_{1}\left(t\right)$, $\beta_{2}\left(t\right)$, $Z_{1}\left(t\right)$, and $Z_{2}\left(t\right)$ obtained from the following set of equations:

(46) $$\begin{array}{ccc} \frac{1}{2\beta_{1}}\frac{d}{dt}\beta_{1}\;\;\; & = & \;\;\;-\frac{2\gamma }{\nu -1}\frac{I_{\nu }}{N_{\nu }^{\nu }N_{\gamma }^{\gamma -1}}\beta_{2}^{(\nu -1)/2}\beta_{1}^{(\gamma +1)/2} \end{array}$$

and

(47) $$\begin{array}{ccc} \frac{1}{2\beta_{2}}\frac{d}{dt}\beta_{2}\;\;\; & = & \;\;\;-\frac{2\nu }{\gamma -1}\frac{I_{\gamma }}{N_{\gamma }^{\gamma }N_{\nu }^{\nu -1}}\beta_{2}^{(\nu +1)/2}\beta_{1}^{(\gamma -1)/2} \end{array}$$

with

(48) $$\begin{array}{c} I_{\kappa }=\left\{\begin{array}{cc} \frac{\Gamma \left(\frac{1}{2}\right)\Gamma \left(1+\frac{\kappa }{\kappa -1}\right)}{\sqrt{\kappa -1}\Gamma \left(\frac{3}{2}+\frac{\kappa }{\kappa -1}\right)} & 1\leq \kappa <2\\ \frac{\Gamma \left(\frac{1}{2}\right)\Gamma \left(\frac{\kappa }{1-\kappa }-\frac{1}{2}\right)}{\sqrt{1-\kappa }\Gamma \left(\frac{\kappa }{1-\kappa }\right)} & 0\leq \kappa \leq 1 \end{array}\right.\;\;,\;\;N_{\kappa }=\left\{\begin{array}{cc} \frac{\Gamma \left(\frac{1}{2}\right)\Gamma \left(1+\frac{1}{\kappa -1}\right)}{\sqrt{\kappa -1}\Gamma \left(\frac{3}{2}+\frac{1}{\kappa -1}\right)} & 1\leq \kappa <2\\ \frac{\Gamma \left(\frac{1}{2}\right)\Gamma \left(\frac{1}{1-\kappa }-\frac{1}{2}\right)}{\sqrt{1-\kappa }\Gamma \left(\frac{1}{1-\kappa }\right)} & 0\leq \kappa \leq 1 \end{array}\right. \end{array}$$

where κ=γ or ν and $Z_{1(2)}\left(t\right)\sqrt{\beta_{1(2)}\left(t\right)}=Z_{1(2)}\left(0\right)\sqrt{\beta_{1(2)}\left(0\right)}=constant$. Note that depending on the choice of the parameters γ and ν, Equations (41) and (42) may present a compact or a long-tailed behavior. In the last case, it is possible to connect the solutions with the Lévy distributions as performed in Refs. [54,55]. The solutions for Equations (46) and (47) can be found, and they are given by

(49) $$\begin{array}{c} \beta_{1}\left(t\right)={\left[\frac{\nu +\gamma }{\nu -1}\frac{\gamma I_{\nu }}{N_{\nu }N_{\gamma }^{\gamma -1}}C^{\frac{\nu -1}{2}}t\right]}^{-\frac{2}{\nu +\gamma }} \end{array}$$

and $\beta_{2}\left(t\right)=C\beta_{1}\left(t\right)$ with

(50) $$\begin{array}{c} C=\frac{\gamma (\gamma -1)}{\nu (\nu -1)}\left(\frac{I_{\nu }N_{\gamma }}{I_{\gamma }N_{\nu }}\right). \end{array}$$

By using the previous equations, it is possible to obtain the mean square displacement. Let us now consider the case of $\xi_{1(2)}\left(x\right)={\left|x\right|}^{\alpha_{1(2)}}$, which represents a fractal metric as proposed by Chen [19], and the definition that follows:

(51) $$\begin{array}{c} \sigma_{x,1(2)}^{2}\left(t\right)=\langle {\left(x_{1(2)}-{\langle x_{1(2)}\rangle }_{1(2)}\right)}^{2}\rangle . \end{array}$$

**Figure 2:**
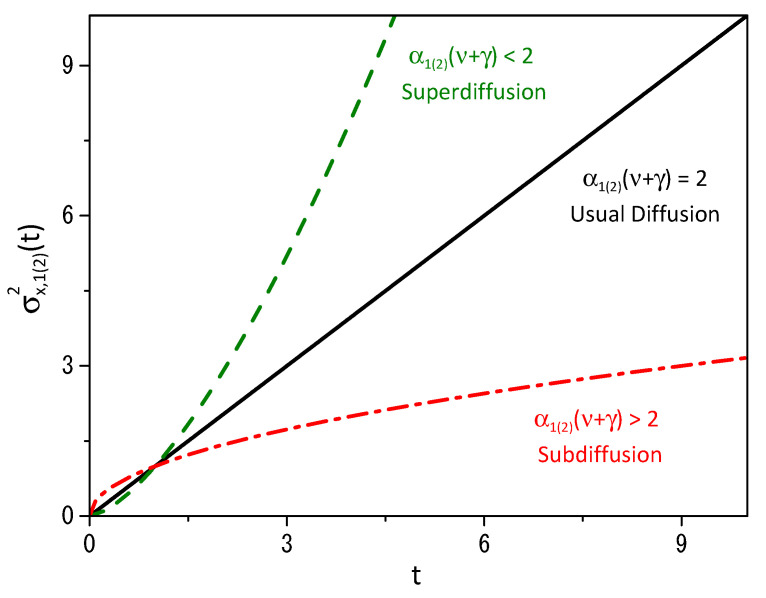
Figure 2 shows the behavior of the mean square displacement obtained for $\rho_{1}\left(x_{1},t\right)$ and $\rho_{2}\left(x_{2},t\right)$ from Equations (3) and (26). The solid black line corresponds to the standard diffusion. The green dashed line corresponds to the superdiffusion. The red dashed-dotted line is associated with subdiffusion. To make things simpler, we assume $\rho_{1(2)}\left(x_{1(2)},0\right)=\delta \left(x_{1(2)}\right)$, $\xi_{I,1(2)}\left(x_{1(2)}\right)={\left|x_{1(2)}\right|}^{\alpha_{1(2)}}$, and D=1.

After performing some calculations, it is possible to show that

(52) $$\begin{array}{c} \sigma_{x,1(2)}^{2}\left(t\right)=\frac{\zeta_{\gamma (\nu )}}{N_{\gamma (\nu )}}\frac{1}{{\left[\beta_{1(2)}\left(t\right)\right]}^{\frac{1}{\alpha_{1(2)}}}}, \end{array}$$

where $\zeta_{\kappa }=\int_{0}^{\infty }duu^{1/\alpha_{1(2)}-1/2}\exp_{\kappa }\left[-u\right]$, which implies in $\sigma_{x,1(2)}^{2}\left(t\right)\propto t^{2/[\alpha_{1(2)}\left(\gamma +\nu \right)]}$. Figure 2 illustrates the behavior of the mean square displacement, where the regions for the sub- and superdiffusion are shown. The black line corresponds to the usual diffusion. The choice of $\alpha_{1}$ and $\alpha_{2}$ connected to the fractal derivative directly influences the diffusion process. Another point concerning the behavior showed in Figure 2 is the influence of the dynamic of each system on the other since the results for the mean square displacement depend on the parameters γ and ν connected to the nonlinearity present in the diffusive term. These feature connected to Figure 2 shows that the anomalous behavior is directly connected with the fractal metric of space [19] and has also been worked out in Refs. [56,57,58,59] by considering diffusion on fractal objects.

**Figure 3:**
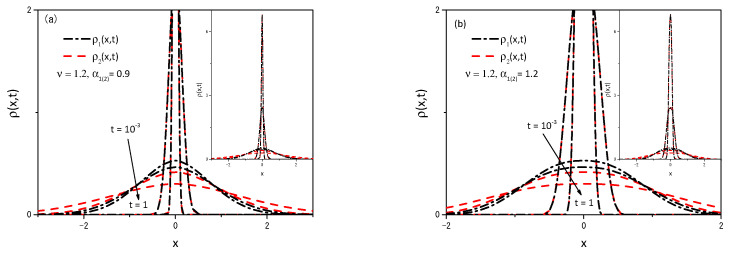
Behavior obtained for $\rho_{1}\left(x_{1},t\right)$ and $\rho_{2}\left(x_{2},t\right)$ from Equations (3) and (6) in Figure 3a and Figure 3b, where $P_{1(2)}\left(\rho_{1(2)},t\right)$ is given by Equation (14) with $p\left(\bar{\gamma }\right)=\delta \left(\bar{\gamma }-1\right)/2+\delta \left(\nu -\bar{\gamma }\right)/2$, $F_{1}\left(x_{1},t\right)=-k_{1}\left(\theta \left(x_{1}\right)-\theta \left(-x_{1}\right)\right){\left|x_{1}\right|}^{\alpha_{1}}$ (θ(x) is the Heviside function), and $F_{2}\left(x_{2},t\right)=0$. The black dashed lines correspond to $\rho_{1}\left(x_{1},t\right)$ for different values of *t*. The red dashed-dotted lines correspond to $\rho_{2}\left(x_{2},t\right)$ for different values of *t*. We consider, for simplicity, $\rho_{1(2)}\left(x_{1(2)},0\right)=\delta \left(x_{1(2)}\right)$, $k_{1}=1$, $\xi_{I,1(2)}\left(x_{1(2)}\right)={\left|x_{1(2)}\right|}^{\alpha_{1(2)}}$, and D=1.

**Figure 4:**
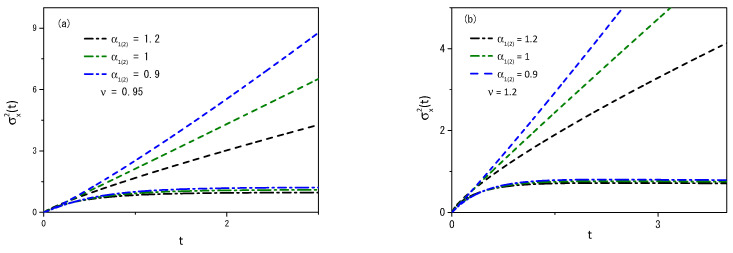
Figure 4a and Figure 4b show the behavior of the mean square displacement obtained with the distributions $\rho_{1}\left(x_{1},t\right)$ and $\rho_{2}\left(x_{2},t\right)$ from Equations (3) and (6), where $P_{1(2)}\left(\rho_{1(2)},t\right)$ is given by Equation (14) with $p\left(\bar{\gamma }\right)=\delta \left(\bar{\gamma }-1\right)/2+\delta \left(\nu -\bar{\gamma }\right)/2$, $F_{1}\left(x_{1},t\right)=-k_{1}\left(\theta \left(x_{1}\right)-\theta \left(-x_{1}\right)\right){\left|x_{1}\right|}^{\alpha_{1}}$ (θ(x) is the Heaviside function) and $F_{2}\left(x_{2},t\right)=0$. The dashed-dotted lines correspond to the mean square displacement obtained from $\rho_{1}\left(x_{1},t\right)$ for different values of ν. dashed lines correspond to the mean square displacement obtained from $\rho_{2}\left(x_{2},t\right)$ for different values of ν. We consider, for simplicity, $\rho_{1(2)}\left(x_{1(2)},0\right)=\delta \left(x_{1(2)}\right)$, $k_{1}=1$, $\xi_{I,1(2)}\left(x_{1(2)}\right)={\left|x_{1(2)}\right|}^{\alpha_{1(2)}}$, and D=1.

It is possible to demonstrate other scenarios by performing numerical computations, that is, by numerically solving Equations (2) and (5). Figure 3 and Figure 4 illustrate the case for which $P_{1(2)}\left(\rho_{1(2)},t\right)$ is given by Equation (14) with $p\left(\bar{\gamma }\right)=\delta \left(\bar{\gamma }-1\right)/2+\delta \left(\nu -\bar{\gamma }\right)/2$, $F_{1}\left(x_{1},t\right)=-k_{1}\left(\theta \left(x_{1}\right)-\theta \left(-x_{1}\right)\right){\left|x_{1}\right|}^{\alpha_{1}}$ (θ(x) is the Heaviside function), and $F_{2}\left(x_{1},t\right)=0$. Notice that the initial moments, with centered distributions, represent $t=10^{-3}$, while the more spread distributions represent t=1. Remarkably, a simple change in the fractal value $\alpha_{1(2)}$ results in a large change in the observed diffusion regime of the particles. Numerical computations were performed for values ν greater than and less than one. The system was set in the range of −5000 to 5000, with a step size of dx=0.02 and a time step of dt=0.000001 to generate the results shown in the figures. The choices of dx and dt meet the requirement of $Ddt/\left(dx^{2}\right)<1/2$ for the stability of the solutions when the initial condition evolves in time to meet the boundary conditions [60,61]. Figure 3 show the dynamical behavior of the distributions $\rho_{1(2)}\left(x,t\right)$ for ν=1.2, and two values of the fractal exponent $\alpha_{1(2)}$, which produce a non-Gaussian result. Note that the behavior presented in Figure 3 results from combining two different diffusive terms, one linear and the other nonlinear, besides the unusual metric for the space. Figure 4 demonstrates the anomalous nature of diffusive behavior when the fractal exponent changes from 0.9 to 1.2 for ν=0.95 and ν=1.2. It is also interesting to mention that the results presented in this figure show that the system under the influence of the external force has a different behavior for the mean square displacement, that is, the influence of the external force limits diffusion. On the other hand, the system without external force can spread freely. The influence is also present on the time dependence of the diffusion coefficient, which depends on the integral of the distribution in a nonlinear power law of the other distribution.

### 2.2. Entropy Production

We can examine the entropy production associated with Equation (21) by looking at the dynamics of $\rho_{1}\left(x_{1},t\right)$ and $\rho_{2}\left(x_{2},t\right)$ given by Equations (25) and (26). Differentiating Equation (21) with respect to time gives us the result:

(53) $$\begin{array}{ccc} \frac{d}{dt}S\left(t\right) & = & k\int_{-\infty }^{\infty }dx_{1}\xi_{1}\left(x_{1}\right)\int_{-\infty }^{\infty }dx_{2}\xi_{2}\left(x_{2}\right)\left[\frac{\partial }{\partial \rho_{12}}s\left(\rho_{12}\right)\right]\frac{\partial }{\partial t}\left[\rho_{1}\left(x_{1},t\right)\rho_{2}\left(x_{2},t\right)\right]\\ & = & -k\int_{-\infty }^{\infty }dx_{1}\xi_{1}\left(x_{1}\right)\int_{-\infty }^{\infty }dx_{2}\xi_{2}\left(x_{2}\right)\rho_{2}\frac{\partial }{\partial \rho_{12}}s\left(\rho_{12}\right)\frac{\partial }{\partial \xi_{I,1}\left(x_{1}\right)}J_{1}\left(x_{1},t\right)\\ & - & k\int_{-\infty }^{\infty }dx_{1}\xi_{1}\left(x_{1}\right)\rho_{1}\int_{-\infty }^{\infty }dx_{2}\xi_{2}\left(x_{2}\right)\frac{\partial }{\partial \rho_{12}}s\left(\rho_{12}\right)\frac{\partial }{\partial \xi_{I,2}\left(x_{2}\right)}J_{2}\left(x_{2},t\right) \end{array}$$

Consequently, integration by parts is performed with the conditions that $J_{1}\left(x_{1}\to \pm \infty ,t\right)$ and $J_{2}\left(x_{2}\to \pm \infty ,t\right)$ both approaching zero:

(54) $$\begin{array}{ccc} \frac{d}{dt}S\left(t\right) & = & k\int_{-\infty }^{\infty }dx_{1}\xi_{1}\left(x_{1}\right)\int_{-\infty }^{\infty }dx_{2}\xi_{2}\left(x_{2}\right)\left[\rho_{2}^{2}\frac{\partial^{2}}{\partial \rho_{12}^{2}}s\left(\rho_{12}\right)\frac{\partial }{\partial \xi_{I,1}\left(x_{1}\right)}\rho_{1}\right]J_{1}\left(x_{1},t\right)\\ & + & k\int_{-\infty }^{\infty }dx_{1}\xi_{1}\left(x_{1}\right)\int_{-\infty }^{\infty }dx_{2}\xi_{2}\left(x_{2}\right)\left[\rho_{1}^{2}\frac{\partial^{2}}{\partial \rho_{12}^{2}}s\left(\rho_{12}\right)\frac{\partial }{\partial \xi_{I,2}\left(x_{2}\right)}\rho_{2}\right]J_{2}\left(x_{2},t\right). \end{array}$$

It is feasible to make Equation (53) simpler by utilizing the equations from the H-Theorem:

(55) $$\begin{array}{c} -kT\rho_{1}\rho_{2}^{2}\frac{\partial \rho_{1}}{\partial \xi_{I,1}\left(x_{1}\right)}\frac{\partial^{2}}{\partial \rho_{12}^{2}}s\left(\rho_{12}\right)=D\frac{\partial }{\partial \xi_{I,1}\left(x_{1}\right)}P_{1}\left(\rho_{1},t\right) \end{array}$$

and

(56) $$\begin{array}{c} -kT\rho_{2}\rho_{1}^{2}\frac{\partial \rho_{2}}{\partial \xi_{I,2}\left(x_{2}\right)}\frac{\partial^{2}}{\partial \rho_{12}^{2}}s\left(\rho_{12}\right)=D\frac{\partial }{\partial \xi_{I,2}\left(x_{2}\right)}P_{2}\left(\rho_{2},t\right), \end{array}$$

to obtain

(57) $$\begin{array}{ccc} \frac{d}{dt}S\left(t\right) & = & -\frac{1}{T}\int_{-\infty }^{\infty }dx_{1}\xi_{1}\left(x_{1}\right)F_{1}\left(x_{1}\right)J_{1}\left(x_{1},t\right)-\frac{1}{T}\int_{-\infty }^{\infty }dx_{2}\xi_{2}\left(x_{2}\right)F_{2}\left(x_{2}\right)J_{2}\left(x_{1},t\right)\\ & + & \frac{1}{T}\int_{-\infty }^{\infty }dx_{1}\xi_{1}\left(x_{1}\right)\frac{J_{1}^{2}\left(x_{1},t\right)}{\rho_{1}\left(x_{1},t\right)}+\frac{1}{T}\int_{-\infty }^{\infty }dx_{2}\xi_{2}\left(x_{2}\right)\frac{J_{2}^{2}\left(x_{2},t\right)}{\rho_{2}\left(x_{2},t\right)}, \end{array}$$

where $J_{1}\left(x_{1},t\right)$ and $J_{2}\left(x_{2},t\right)$ are defined by Equations (1) and (5), with $P_{1}\left(\rho_{1},t\right)$ and $P_{2}\left(\rho_{2},t\right)$ given by Equations (30) and (31). Equation (53) can also be written as follows:

(58) $$\begin{array}{c} \frac{d}{dt}S=\Pi -\Phi . \end{array}$$

The entropy that is exchanged between the two subsystems $\rho_{1}$ and $\rho_{2}$ and their environment is called the flux of entropy. This can be expressed as follows:

(59) $$\begin{array}{c} \Phi =\frac{1}{T}\int_{-\infty }^{\infty }dx_{1}\xi_{1}\left(x_{1}\right)F_{1}\left(x_{1}\right)J_{1}\left(x_{1},t\right)+\frac{1}{T}\int_{-\infty }^{\infty }dx_{2}\xi_{2}\left(x_{1}\right)F_{2}\left(x_{2}\right)J_{2}\left(x_{1},t\right), \end{array}$$

and the entropy-production term:

(60) $$\begin{array}{c} \Pi =\frac{1}{T}\int_{-\infty }^{\infty }dx_{1}\xi_{1}\left(x_{1}\right)\frac{J_{1}^{2}\left(x_{1},t\right)}{\rho_{1}\left(x_{1},t\right)}+\frac{1}{T}\int_{-\infty }^{\infty }dx_{2}\xi_{2}\left(x_{2}\right)\frac{J_{2}^{2}\left(x_{2},t\right)}{\rho_{2}\left(x_{2},t\right)}. \end{array}$$

Since *T* and $\rho_{i}\left(x_{i},t\right)$ are both positive, the desired result is obtained: Π≥0. This result for the entropy production, given by Equation (57) and, thus, Equation (58) can also be confirmed for any entropy condition.

**Figure 5:**
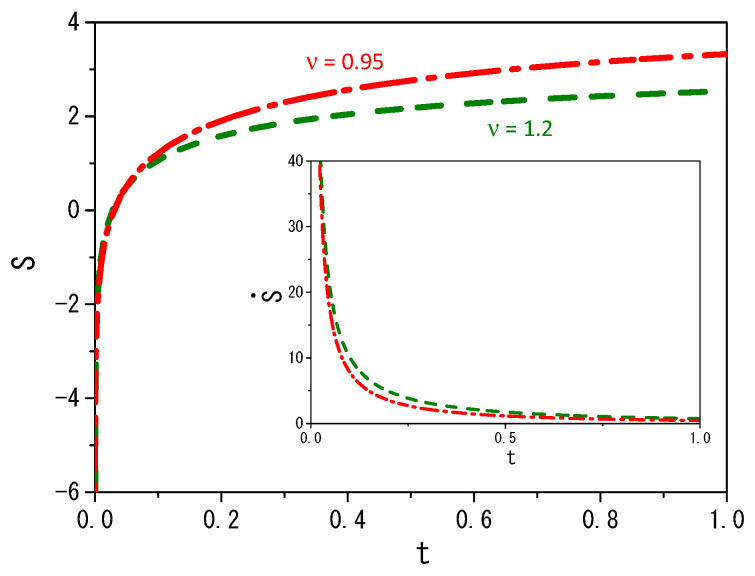
Figure 5 shows the behavior obtained for Equation (22) with $\rho_{1}\left(x_{1},t\right)$ and $\rho_{2}\left(x_{2},t\right)$ from Equations (3) and (6), where $P_{1(2)}\left(\rho_{1(2)},t\right)$ is given by Equation (14) with $p\left(\bar{\gamma }\right)=\delta \left(\bar{\gamma }-1\right)/2+\delta \left(\nu -\bar{\gamma }\right)/2$, $F_{1}\left(x_{1},t\right)=-k_{1}\left(\theta \left(x_{1}\right)-\theta \left(-x_{1}\right)\right){\left|x_{1}\right|}^{\alpha_{1}}$ (θ(x) is the Heviside function), and $F_{2}\left(x_{2},t\right)=0$. The red dashed-dotted line corresponds to the case ν=0.9. The green dashed line corresponds to the case ν=1.2. The inset corresponds to the behavior of Equation (57) for these values of ν. We consider, for simplicity, $\rho_{1(2)}\left(x_{1(2)},0\right)=\delta \left(x_{1(2)}\right)$, $k_{1}=1$, $\xi_{I,1(2)}\left(x_{1(2)}\right)={\left|x_{1(2)}\right|}^{\alpha_{1(2)}}$, and D=1.

**Figure 6:**
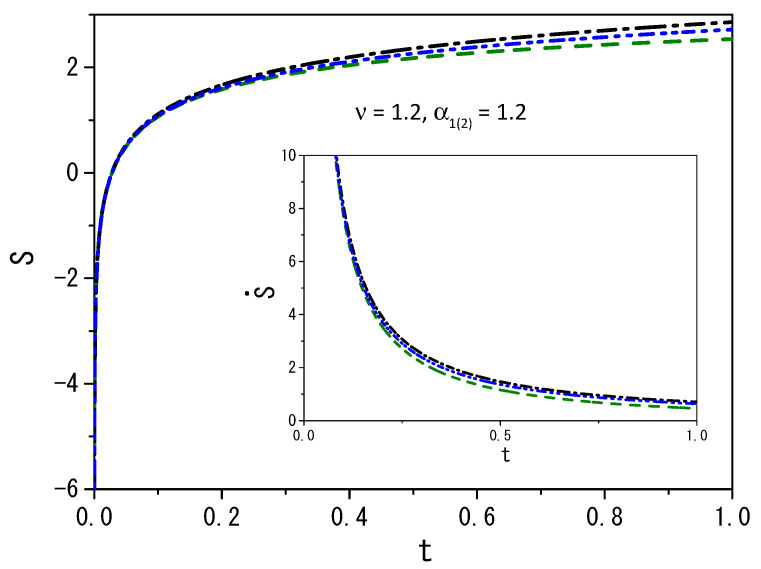
Figure 6 shows the behavior obtained for Equation (22) with $\rho_{1}\left(x_{1},t\right)$ and $\rho_{2}\left(x_{2},t\right)$ from Equations (3) and (6), where $P_{1(2)}\left(\rho_{1(2)},t\right)$ is given by Equation (14) with $p\left(\bar{\gamma }\right)=\delta \left(\bar{\gamma }-1\right)/2+\delta \left(\nu -\bar{\gamma }\right)/2$. The black dashed-dotted line corresponds to the case $F_{1}\left(x_{1},t\right)=0$ and $F_{2}\left(x_{2},t\right)=0$. The green dashed line corresponds to the case $F_{1}\left(x_{1},t\right)=-k_{1}\left(\theta \left(x_{1}\right)-\theta \left(-x_{1}\right)\right){\left|x_{1}\right|}^{\alpha_{1}}$ (θ(x) is the Heviside function), and $F_{2}\left(x_{2},t\right)=0$. The blue dashed-dotted-dotted line corresponds to the case $F_{1}\left(x_{1},t\right)=-k_{1}\left(\theta \left(x_{1}\right)-\theta \left(-x_{1}\right)\right){\left|x_{1}\right|}^{\alpha_{1}}/\left(1+|x_{1}{|}^{2\alpha_{1}}\right)$, and $F_{2}\left(x_{2},t\right)=0$. For simplicity, we consider $\rho_{1(2)}\left(x_{1(2)},0\right)=\delta \left(x_{1(2)}\right)$, $k_{1}=1$, $\xi_{I,1(2)}\left(x_{1(2)}\right)={\left|x_{1(2)}\right|}^{\alpha_{1(2)}}$, and D=1.

It is also important to mention that the entropy production in this framework for the external force considered here has the same behavior for the different choices of $\xi_{I,1(2)}\left(x_{1(2)}\right)$. This feature is directly connected with the definition of the integral used to obtain the entropy. Figure 5 and Figure 6 illustrate the behavior of entropy (S) and entropy production $\left(\dot{S}\right)$. Figure 5 presents (S) and $\left(\dot{S}\right)$ for different values of ν. Figure 6 illustrates the behavior (S) and $\left(\dot{S}\right)$ for the values $\alpha_{1(2)}=1.2$ and ν=1.2 taking into account different external forces. Note that in each case, we consider a diffusion process with two different diffusive terms, one linear and the other nonlinear. Thus, the entropy evaluated for these cases results from the combination of different diffusive regimes. For ν<1, the system is essentially governed by long-tailed distributions, and for ν>1, short-tailed distributions govern the system. These features have a direct influence on entropy and entropy production, as shown in these figures. The entropy of the system is a growing function of time that eventually reaches a plateau for a long time, as expected. Interestingly, the external force, as shown in Figure 6, modifies the system’s entropy. In fact, entropy and entropy production depend on the external force acting on the systems, that is, how it can confine the system or not, during the particle spreading process.

## 3. Conclusions

We have investigated entropy production in a fractal system composed of two subsystems, each subject to an external force. This is achieved by using the H-theorem on the nonlinear Fokker–Planck equations (NFEs), characterizing the diffusing dynamics of each subsystem. To consider the metric of space in which the systems are embedded and, hence, the fractal nature that leads to anomalous diffusion, we expressed the general NFE in terms of Hausdorff derivatives. We investigated some solutions from an analytical and numerical point of view. From our results, it is clear that the diffusive regime is directly related to the system metric, meaning that the distributions characterize anomalous diffusion, which may represent usual, sub-, or super-diffusive processes. It is also interesting to note that each system has an influence on the spread of the other through the diffusive term and the external forces applied to the systems. In this manner, the results have shown that the dynamic of each one has a direct influence on the other as a thermal bath, since the coupling appears in the diffusive term, which is related to how the system will spread. Our results, as they have been obtained, represent a very general approach to describe particle dynamics and thermodynamic connection in systems composed of interacting particles, such as diffusion in crowded biological media [26] and many others. In particular, our results help to demonstrate how entropy production occurs in such systems which, in turn, may be fundamental to understanding several aspects of interacting particle systems and their connection with measurable quantities. Furthermore, the generalization proposed in this work allows one or both subsystems to be fractal in nature, that is, the space morphology is also present within the model. On the other hand, our results indicate that the space metric does not affect the entropy production of the system. Instead, it is directly affected by other aspects of the subsystems related to the intrinsic nature of diffusion. We anticipate that our findings will contribute to a better understanding of the connection between complex systems, nonlinear sciences, and the metric of space in which the process takes place.

## Data Availability

The data presented in this study are available in this article.

## Appendix A

Let us consider in detail the calculations of some equations. We start with the results obtained for Equation (13). For this, we consider the following Equation (A1),

(A1) $$\begin{array}{c} \frac{d}{dt}F=\int_{-\infty }^{\infty }dx_{1}\xi_{1}\left(x_{1}\right)\int_{-\infty }^{\infty }dx_{2}\xi_{2}\left(x_{2}\right)\left[\Xi \left(x_{1},x_{2}\right)-kT\frac{\partial }{\partial \rho_{12}}s\left(\rho_{12}\right)\right]\frac{\partial }{\partial t}\left[\rho_{1}\left(x_{1},t\right)\rho_{2}\left(x_{2},t\right)\right], \end{array}$$

in which $\rho_{12}=\rho_{1}\rho_{2}$, as defined before. After performing the time derivative, Equation (A1) can be written as follows

(A2) $$\begin{array}{ccc} \frac{d}{dt}F & = & \int_{-\infty }^{\infty }dx_{1}\xi_{1}\left(x_{1}\right)\int_{-\infty }^{\infty }dx_{2}\xi_{2}\left(x_{2}\right)\left[\Xi \left(x_{1},x_{2}\right)-kT\frac{\partial }{\partial \rho_{12}}s\left(\rho_{12}\right)\right]\\ & \times & \left[\rho_{2}\left(x_{2},t\right)\frac{\partial }{\partial t}\rho_{1}\left(x_{1},t\right)+\rho_{1}\left(x_{1},t\right)\frac{\partial }{\partial t}\rho_{2}\left(x_{2},t\right)\right]\;. \end{array}$$

Now, we need to consider the nonlinear Fokker–Planck equations, i.e., Equations (3) and (6), to simplify the differential operators in time. By substituting Equations (3) and (6) in Equation (A2), we have that

(A3) $$\begin{array}{ccc} \frac{d}{dt}F & = & \int_{-\infty }^{\infty }dx_{1}\xi_{1}\left(x_{1}\right)\int_{-\infty }^{\infty }dx_{2}\xi_{2}\left(x_{2}\right)\left\{\Xi \left(x_{1},x_{2}\right)\rho_{2}-kT\rho_{2}\frac{\partial }{\partial \rho_{12}}s\left(\rho_{12}\right)\right\}\\ & \times & \frac{\partial }{\partial \xi_{I,1}\left(x_{1}\right)}\left\{D\frac{\partial }{\partial \xi_{I,1}\left(x_{1}\right)}P_{1}\left(\rho_{1},t\right)-\frac{\partial }{\partial \xi_{I,1}\left(x_{1}\right)}\left[F_{1}\left(x_{1}\right)\rho_{1}\left(x_{1},t\right)\right]\right\}\\ & + & \int_{-\infty }^{\infty }dx_{1}\xi_{1}\left(x_{1}\right)\int_{-\infty }^{\infty }dx_{2}\xi_{2}\left(x_{2}\right)\left\{\Xi \left(x_{1},x_{2}\right)\rho_{1}-kT\rho_{1}\frac{\partial }{\partial \rho_{12}}s\left(\rho_{12}\right)\right\}\\ & \times & \frac{\partial }{\partial \xi_{I,2}\left(x_{2}\right)}\left\{D\frac{\partial }{\partial \xi_{I,2}\left(x_{2}\right)}P_{2}\left(\rho_{2},t\right)-\frac{\partial }{\partial \xi_{I,2}\left(x_{2}\right)}\left[F_{2}\left(x_{2}\right)\rho_{2}\left(x_{2},t\right)\right]\right\}. \end{array}$$

Before performing integration by parts, we can use the properties of the fractal derivative and write the previous equation as follows:

(A4) $$\begin{array}{ccc} \frac{d}{dt}F & = & \int_{-\infty }^{\infty }dx_{2}\xi_{2}\left(x_{2}\right)\int_{-\infty }^{\infty }dx_{1}\left\{\Xi \left(x_{1},x_{2}\right)\rho_{2}-kT\rho_{2}\frac{\partial }{\partial \rho_{12}}s\left(\rho_{12}\right)\right\}\\ & \times & \frac{\partial }{\partial x_{1}}\left\{D\frac{\partial }{\partial \xi_{I,1}\left(x_{1}\right)}P_{1}\left(\rho_{1},t\right)-\frac{\partial }{\partial \xi_{I,1}\left(x_{1}\right)}\left[F_{1}\left(x_{1}\right)\rho_{1}\left(x_{1},t\right)\right]\right\}\\ & + & \int_{-\infty }^{\infty }dx_{1}\xi_{1}\left(x_{1}\right)\int_{-\infty }^{\infty }dx_{2}\left\{\Xi \left(x_{1},x_{2}\right)\rho_{1}-kT\rho_{1}\frac{\partial }{\partial \rho_{12}}s\left(\rho_{12}\right)\right\}\\ & \times & \frac{\partial }{\partial x_{2}}\left\{D\frac{\partial }{\partial \xi_{I,2}\left(x_{2}\right)}P_{2}\left(\rho_{2},t\right)-\frac{\partial }{\partial \xi_{I,2}\left(x_{2}\right)}\left[F_{2}\left(x_{2}\right)\rho_{2}\left(x_{2},t\right)\right]\right\}. \end{array}$$

We perform some integration by parts and assume the following conditions: $\rho_{i}\left(x\to \pm \infty ,t\right)\to 0$ and $\partial_{\xi_{I,i}\left(x_{i}\right)}\rho_{i}\left(x_{i},t\right){|}_{x_{i}\to \pm \infty }\to 0$. Thus, Equation (A4) yields:

(A5) $$\begin{array}{ccc} \frac{d}{dt}F & = & -\int_{-\infty }^{\infty }dx_{2}\xi_{2}\left(x_{2}\right)\int_{-\infty }^{\infty }dx_{1}\frac{\partial }{\partial x_{1}}\left\{\Xi \left(x_{1},x_{2}\right)\rho_{2}-kT\rho_{2}\frac{\partial }{\partial \rho_{12}}s\left(\rho_{12}\right)\right\}\\ & \times & \left\{D\frac{\partial }{\partial \xi_{I,1}\left(x_{1}\right)}P_{1}\left(\rho_{1},t\right)-\frac{\partial }{\partial \xi_{I,1}\left(x_{1}\right)}\left[F_{1}\left(x_{1}\right)\rho_{1}\left(x_{1},t\right)\right]\right\}\\ & - & \int_{-\infty }^{\infty }dx_{1}\xi_{1}\left(x_{1}\right)\int_{-\infty }^{\infty }dx_{2}\frac{\partial }{\partial x_{2}}\left\{\Xi \left(x_{1},x_{2}\right)\rho_{1}-kT\rho_{1}\frac{\partial }{\partial \rho_{12}}s\left(\rho_{12}\right)\right\}\\ & \times & \left\{D\frac{\partial }{\partial \xi_{I,2}\left(x_{2}\right)}P_{2}\left(\rho_{2},t\right)-\frac{\partial }{\partial \xi_{I,2}\left(x_{2}\right)}\left[F_{2}\left(x_{2}\right)\rho_{2}\left(x_{2},t\right)\right]\right\}. \end{array}$$

and, consequently,

(A6) $$\begin{array}{ccc} \frac{d}{dt}F & = & -\int_{-\infty }^{\infty }dx_{1}\xi_{1}\left(x_{1}\right)\int_{-\infty }^{\infty }dx_{2}\xi_{2}\left(x_{2}\right)\left\{\frac{\partial \phi_{1}\left(x_{1}\right)}{\partial \xi_{I,1}\left(x_{1}\right)}\rho_{2}-kT\rho_{2}^{2}\frac{\partial \rho_{1}}{\partial \xi_{I,1}\left(x_{1}\right)}\frac{\partial^{2}}{\partial \rho_{12}^{2}}s\left(\rho_{12}\right)\right\}\\ & \times & \left\{D\frac{\partial }{\partial \xi_{I,1}\left(x_{1}\right)}P_{1}\left(\rho_{1},t\right)+\frac{\partial \phi_{1}\left(x_{1}\right)}{\partial \xi_{I,1}\left(x_{1}\right)}\rho_{1}\left(x_{1},t\right)\right\}\\ & - & \int_{-\infty }^{\infty }dx_{1}\xi_{1}\left(x_{1}\right)\int_{-\infty }^{\infty }dx_{2}\xi_{2}\left(x_{2}\right)\left\{\frac{\partial \phi_{2}\left(x_{2}\right)}{\partial \xi_{I,2}\left(x_{2}\right)}\rho_{1}-kT\rho_{1}^{2}\frac{\partial \rho_{2}}{\partial \xi_{I,2}\left(x_{2}\right)}\frac{\partial^{2}}{\partial \rho_{12}^{2}}s\left(\rho_{12}\right)\right\}\\ & \times & \left\{D\frac{\partial }{\partial \xi_{I,2}\left(x_{2}\right)}P_{2}\left(\rho_{2},t\right)+\frac{\partial \phi_{2}\left(x_{2}\right)}{\partial \xi_{I,2}\left(x_{2}\right)}\rho_{2}\left(x_{2},t\right)\right\}. \end{array}$$

Note that to obtain Equation (A7), i.e.,

(A7) $$\begin{array}{ccc} \frac{d}{dt}F & = & -\int_{-\infty }^{\infty }dx_{1}\xi_{1}\left(x_{1}\right)\frac{1}{\rho_{1}}\\ & \times & {\left\{\int_{-\infty }^{\infty }dx_{2}\xi_{2}\left(x_{2}\right)\left[\frac{\partial \phi_{1}\left(x_{1}\right)}{\partial \xi_{I,1}\left(x_{1}\right)}\rho_{2}\rho_{1}-kT\rho_{2}^{2}\rho_{1}\frac{\partial \rho_{1}}{\partial \xi_{I,1}\left(x_{1}\right)}\frac{\partial^{2}}{\partial \rho_{12}^{2}}s\left(\rho_{12}\right)\right]\right\}}^{2}\\ & - & \int_{-\infty }^{\infty }dx_{2}\xi_{2}\left(x_{2}\right)\frac{1}{\rho_{2}}\\ & \times & {\left\{\int_{-\infty }^{\infty }dx_{1}\xi_{1}\left(x_{1}\right)\left[\frac{\partial \phi_{2}\left(x_{2}\right)}{\partial \xi_{I,2}\left(x_{2}\right)}\rho_{1}\rho_{2}-kT\rho_{1}^{2}\rho_{2}\frac{\partial \rho_{2}}{\partial \xi_{I,2}\left(x_{2}\right)}\frac{\partial^{2}}{\partial \rho_{12}^{2}}s\left(\rho_{12}\right)\right]\right\}}^{2}. \end{array}$$

by taking Equations (30) and (31) into account, we have to consider

(A8) $$\begin{array}{ccc} -kT\rho_{j}^{2}\rho_{i}\frac{\partial^{2}}{\partial \rho_{ij}^{2}}s\left(\rho_{ij}\right) & = & D\rho_{j}\frac{\partial }{\partial \rho_{ij}}\left(\int_{0}^{\gamma }d\bar{\gamma }p\left(\bar{\gamma }\right)\rho_{i}^{\bar{\gamma }}\rho_{j}^{\bar{\gamma }}\right)\;, \end{array}$$

(A9) $$\begin{array}{ccc} & = & D\rho_{j}\frac{\partial }{\partial \rho_{ij}}\left(\int_{0}^{\gamma }d\bar{\gamma }p\left(\bar{\gamma }\right)\rho_{ij}^{\bar{\gamma }}\right)\;, \end{array}$$

where i=1,2 and j=1,2 with i≠j, D=kT, $\rho_{ij}=\rho_{i}\rho_{j}$, and $\int_{-\infty }^{\infty }dx_{i}\xi_{i}\left(x_{i}\right)\rho_{i}\left(x_{i},t\right)=1$, which implies

(A10) $$\begin{array}{c} \frac{d}{dt}F\leq 0\;. \end{array}$$

Similar calculations were performed to obtain Equations (29)–(38).
